# Supplementary material for: CDK-regulated dimerization of M18BP1 on a Mis18 hexamer is necessary for CENP-A loading
Source: eLife. 2017 Jan 6;6:e23352. doi: 10.7554/eLife.23352 (PMC5245964; doi:10.7554/eLife.23352)
Supplement: Supplementary file 1. — DOI: http://dx.doi.org/10.7554/eLife.23352.018 [file elife-23352-supp1.doc]

**Supplementary File 1. Plasmid vectors used in this study**

| **Plasmid name** | **Parental vector** | **Reference** |
| --- | --- | --- |
| pETDuet-1 |  | Novagen |
| pETDuet-8His | pETDuet-1 | This study |
| pETDuet-MBP-8His | pETDuet-8His | This study |
| pETDuet-MBP-M18BP1(1-140)-8His | pETDuet-MBP-8His | This study |
| pETDuet-MBP-M18BP1(1-60)-8His | pETDuet-MBP-8His | This study |
| pETDuet-MBP-M18BP1(61-140)-8His | pETDuet-MBP-8His | This study |
| pETDuet-MBP-M18BP1(1-140/T40D/S110E)-8His | pETDuet-MBP-M18BP1(1-140)-8His | This study |
| pETDuet-MBP-M18BP1(1-140/T40V/S110A)-8His | pETDuet-MBP-M18BP1(1-140)-8His | This study |
| pETDuet-MBP-M18BP1(1-60/T4D)-8His | pETDuet-MBP-M18BP1(1-60)-8His | This study |
| pETDuet-MBP-M18BP1(1-60/T4E)-8His | pETDuet-MBP-M18BP1(1-60)-8His | This study |
| pETDuet-MBP-M18BP1(1-60/T4V)-8His | pETDuet-MBP-M18BP1(1-60)-8His | This study |
| pETDuet-MBP-M18BP1(1-60/T40D)-8His | pETDuet-MBP-M18BP1(1-60)-8His | This study |
| pETDuet-MBP-M18BP1(1-60/T40E)-8His | pETDuet-MBP-M18BP1(1-60)-8His | This study |
| pETDuet-MBP-M18BP1(1-60/T40V)-8His | pETDuet-MBP-M18BP1(1-60)-8His | This study |
| pETDuet-MBP-M18BP1(1-60/T4V/T40V)-8His | pETDuet-MBP-M18BP1(1-60)-8His | This study |
| pETDuet-MBP-M18BP1(61-140/S110D)-8His | pETDuet-MBP-M18BP1(61-140)-8His | This study |
| pETDuet-MBP-M18BP1(61-140/S110E)-8His | pETDuet-MBP-M18BP1(61-140)-8His | This study |
| pETDuet-MBP-M18BP1(61-140/S110A)-8His | pETDuet-MBP-M18BP1(61-140)-8His | This study |
| pETDuet-6His-Mis18α-MBP-Mis18β | pETDuet-1 | This study |
| pETDuet-6His-Mis18α(1-191)-MBP-Mis18β(1-189) | pETDuet-1 | This study |
| pETDuet-6His-Mis18α(78-191)-MBP-Mis18β(73-189) | pETDuet-1 | This study |
| pETDuet-6His-Mis18α(192-233)- MBP-Mis18β(190-229) | pETDuet-1 | This study |
| pETDuet-6His-Mis18α(192-233)-mCherry- MBP-Mis18β(190-229) | pETDuet-6His-Mis18α(192-233)- MBP-Mis18β(190-229) | This study |
| pETDuet-6His-Mis18α(78-191) | pETDuet-1 | This study |
| pETDuet-6His-Mis18β(73-189) | pETDuet-1 | This study |
| pETDuet-6His-MBP-Mis18α | pETDuet-1 | This study |
| pETDuet-6His-MBP-Mis18β | pETDuet-1 | This study |
| pGEX-6P-1 |  | GE Healthcare |
| pGEX6PT-M18BP1(1-1132)-MBP | pGEX-6P-1 | This study |
| pGEX6PT-M18BP1(1-490)-MBP | pGEX6PT-M18BP1(1-1132)-MBP | This study |
| pGEX6PT-M18BP1(491-1132)-MBP | pGEX6PT-M18BP1(1-1132)-MBP | This study |
| pGEX6PT-M18BP1(1-228)-MBP | pGEX6PT-M18BP1(1-1132)-MBP | This study |
| pGEX6PT-M18BP1(229-490)-MBP | pGEX6PT-M18BP1(1-1132)-MBP | This study |
| pGEX6PT-M18BP1(1-140)-MBP | pGEX6PT-M18BP1(1-1132)-MBP | This study |
| pGEX6PT-M18BP1(141-228)-MBP | pGEX6PT-M18BP1(1-1132)-MBP | This study |
| pGEX6PT-M18BP1(141-1132)-MBP | pGEX6PT-M18BP1(1-1132)-MBP | This study |
| pLIB |  | (Weissmann et al., 2016) |
| pLIB-6His-Mis18α | pLIB | This study |
| pLIB-MBP-Mis18α | pLIB | This study |
| pLIB-Mis18β | pLIB | This study |
| pLIB-6His-Mis18β | pLIB | This study |
| pLIB-MBP-Mis18β | pLIB | This study |
| pBIG1a |  | (Weissmann et al., 2016) |
| pBIG1a -GST-CDK1-6His-cyclin-B1 | pBIG1a | This study |
| pBIG1e |  | (Weissmann et al., 2016) |
| pBIG1e-6His-Mis18α-Mis18β | pBIG1e | This study |
| pBIG1e-6His-Mis18α-MBP-Mis18β | pBIG1e | This study |
| pBIG1e-6His-Mis18β-MBP-Mis18α | pBIG1e | This study |
| pOG44 |  | Thermo Fisher  Scientific |
| pcDNA5/FRT/TO |  | Thermo Fisher Scientific |
| pcDNA5-EGFP-NLS-P2A-mCherry-PTS1 | pcDNA5/FRT/TO | This study |
| pcDNA5-EGFP-NLS-T2A-mCherry-PTS1 | pcDNA5/FRT/TO | This study |
| pcDNA5-EGFP-NLS-P2AT2A-mCherry-PTS1 | pcDNA5/FRT/TO | This study |
| pcDNA5-MTS-TagBFP-P2AT2A-EGFP-NLS-  P2AT2A-mCherry-PTS1 | pcDNA5-EGFP-NLS-P2AT2A-  mCherry-PTS1 | This study |
| pcDNA5-EGFP-M18BP1(1-1132)-P2AT2A-  mCherry-Mis18α | pcDNA5-EGFP-NLS-P2AT2A-  mCherry-PTS1 | This study |
| pcDNA5-EGFP-M18BP1(1-140)-P2AT2A-  mCherry-Mis18α | pcDNA5-EGFP-M18BP1(1-1132)-  P2AT2A-mCherry-Mis18α | This study |
| pcDNA5-EGFP-M18BP1(141-1132)-P2AT2A-  mCherry-Mis18α | pcDNA5-EGFP-M18BP1(1-1132)-  P2AT2A-mCherry-Mis18α | This study |
| pcDNA5-EGFP-M18BP1(1-1132/T40D/S110E)- P2AT2A-mCherry-Mis18α | pcDNA5-EGFP-M18BP1(1-1132)-  P2AT2A-mCherry-Mis18α | This study |
| pcDNA5-EGFP-M18BP1(1-140/T40D/S110E)- P2AT2A-mCherry-Mis18α | pcDNA5-EGFP-M18BP1(1-140)-  P2AT2A-mCherry-Mis18α | This study |
| pcDNA5-GST-EGFP-M18BP1(1-1132)-  P2AT2A-mCherry-Mis18α | pcDNA5-EGFP-M18BP1(1-1132)-  P2AT2A-mCherry-Mis18α | This study |
| pcDNA5-GST-EGFP-M18BP1(141-1132)-  P2AT2A-mCherry-Mis18α | pcDNA5-EGFP-M18BP1(141-1132)-  P2AT2A-mCherry-Mis18α | This study |
| pcDNA5-EGFP-M18BP1(1-140)-P2AT2A-  mCherry-EGFP-M18BP1(1-140) | pcDNA5-EGFP-M18BP1(1-140)-P2AT2A-  mCherry-Mis18α | This study |
| pcDNA5-EGFP-M18BP1(1-140/T40D/S110E)- P2AT2A-mCherry-M18BP1(1-140/T40D/S110E) | pcDNA5-EGFP-M18BP1(1-140/T40D/S110E)- P2AT2A-mCherry-Mis18α | This study |
| pcDNA5-GST-EGFP-M18BP1(1-140)-P2AT2A-  GST-mCherry-EGFP-M18BP1(1-140) | pcDNA5-EGFP-M18BP1(1-140)-P2AT2A-  mCherry-M18BP1(1-140) | This study |
| pcDNA5-GST-EGFP-M18BP1(1-140/T40D/S110E)-P2AT2A-GST-mCherry-M18BP1(1-140/T40D/S110E) | pcDNA5-EGFP-M18BP1(1-140/T40D/S110E)- P2AT2A-mCherry-M18BP1(1-140/T40D/S110E) | This study |
| pSS26m/CENP-A-SNAP-3HA |  | (Jansen et al., 2007) |
| pETDuet-CENP-A-SNAP-HA-PGK-NeoR | pETDuet-1 | This study |
| pX330 |  | (Ran et al., 2013) |
| pX330-CENP-A-sgRNA | pX330 | This study |
