# Supplementary material for: CDK-regulated dimerization of M18BP1 on a Mis18 hexamer is necessary for CENP-A loading
Source: eLife. 2017 Jan 6;6:e23352. doi: 10.7554/eLife.23352 (PMC5245964; doi:10.7554/eLife.23352)
Supplement: Supplementary file 2. — DOI: http://dx.doi.org/10.7554/eLife.23352.019 [file elife-23352-supp2.doc]

**Supplementary File 2. HeLa cell lines used in this study**

| **Cell line name** | **Transfected vector / parental cell line** | **Reference** |
| --- | --- | --- |
| Flp-In T-REx HeLa |  | Tighe et al., 2008 |
| HeLa CENP-A-SNAP | pX330-CENP-A-sgRNA and a DNA fragment for recombination (SNAP-3HA-PGK-NeoR)  / Flp-In T-REx HeLa | This study |
| HeLa CENP-A-SNAP + EGFP-M18BP1^1-1132^-P2A-T2A-mCherry-Mis18α | pcDNA5-EGFP-M18BP1(1-1132)-P2AT2A-  mCherry-Mis18α / HeLa CENP-A-SNAP | This study |
| HeLa CENP-A-SNAP + EGFP-M18BP1^1-140^-P2A-T2A-mCherry-Mis18α | pcDNA5-EGFP-M18BP1(1-140)-P2AT2A-  mCherry-Mis18α / HeLa CENP-A-SNAP | This study |
| HeLa CENP-A-SNAP + EGFP-M18BP1^141-1132^-P2A-T2A-mCherry-Mis18α | pcDNA5-EGFP-M18BP1(141-1132)-P2AT2A-  mCherry-Mis18α / HeLa CENP-A-SNAP | This study |
| HeLa CENP-A-SNAP + EGFP-M18BP1^1-1132/T40D/S110E^-P2A-T2A  -mCherry-Mis18α | pcDNA5-EGFP-M18BP1(1-1132/T40D/S110E)- P2AT2A-mCherry-Mis18α  / HeLa CENP-A-SNAP | This study |
| HeLa CENP-A-SNAP + EGFP-M18BP1^1-140/T40D/S110E^-P2A-T2A  -mCherry-Mis18α | pcDNA5-EGFP-M18BP1(1-140/T40D/S110E)- P2AT2A-mCherry-Mis18α  / HeLa CENP-A-SNAP | This study |
| HeLa CENP-A-SNAP + GST-EGFP-M18BP1^1-1132^-P2A-T2A  -mCherry-Mis18α | pcDNA5-GST-EGFP-M18BP1(1-1132)-  P2AT2A-mCherry-Mis18α  / HeLa CENP-A-SNAP | This study |
| HeLa CENP-A-SNAP + GST-EGFP-M18BP1^141-1132^-P2A-T2A  -mCherry-Mis18α | pcDNA5-GST-EGFP-M18BP1(141-1132)-  P2AT2A-mCherry-Mis18α  / HeLa CENP-A-SNAP | This study |
| HeLa CENP-A-SNAP + EGFP-M18BP1^1-140^-P2A-T2A-mCherry-M18BP1^1-140^ | pcDNA5-EGFP-M18BP1(1-140)-P2AT2A-  mCherry-EGFP-M18BP1(1-140)  / HeLa CENP-A-SNAP | This study |
| HeLa CENP-A-SNAP + EGFP-M18BP1^1-140/T40D/S110E^-P2A-T2A  -mCherry-M18BP1^1-140/T40D/S110E^ | pcDNA5-EGFP-M18BP1(1-140/T40D/S110E)- P2AT2A-mCherry-M18BP1(1-140/T40D/S110E)  / HeLa CENP-A-SNAP | This study |
| HeLa CENP-A-SNAP + GST-EGFP-M18BP1^1-140^-P2A-T2A  -GST-mCherry-M18BP1^1-140^ | pcDNA5-GST-EGFP-M18BP1(1-140)-P2AT2A-  GST-mCherry-EGFP-M18BP1(1-140)  / HeLa CENP-A-SNAP | This study |
| HeLa CENP-A-SNAP + GST-EGFP-M18BP1^1-140/T40D/S110E^-P2A-T2A  -GST-mCherry-M18BP1^1-140/T40D/S110E^ | pcDNA5-GST-EGFP-M18BP1(1-140/T40D/S110E)-P2AT2A-GST-mCherry-M18BP1(1-140/T40D/S110E) / HeLa CENP-A-SNAP | This study |
| Flp-In T-REx HeLa + EGFP-NLS-P2A-mCherry-PTS1 | pcDNA5-EGFP-NLS-P2A-mCherry-PTS1  / Flp-In T-REx HeLa | This study |
| Flp-In T-REx HeLa + EGFP-NLS-T2A-mCherry-PTS1 | pcDNA5-EGFP-NLS-T2A-mCherry-PTS1  / Flp-In T-REx HeLa | This study |
| Flp-In T-REx HeLa + EGFP-NLS-P2A-T2A-mCherry-PTS1 | pcDNA5-EGFP-NLS-P2AT2A-mCherry-PTS1  / Flp-In T-REx HeLa | This study |
| Flp-In T-REx HeLa + MTS-TagBFP-P2A-T2A-EGFP-NLS-  P2AT2A-mCherry-PTS1 | pcDNA5-MTS-TagBFP-P2AT2A-EGFP-NLS-  P2AT2A-mCherry-PTS1  / Flp-In T-REx HeLa | This study |
